# IgE Mediated Shellfish Allergy in Children—A Review

**DOI:** 10.3390/nu15143112

**Published:** 2023-07-12

**Authors:** Arianna Giannetti, Andrea Pession, Irene Bettini, Giampaolo Ricci, Giuliana Giannì, Carlo Caffarelli

**Affiliations:** 1 Paediatrics Unit, IRCCS Azienda Ospedaliero-Universitaria di Bologna, 40138 Bologna, Italy; ariannagiannetti25@gmail.com (A.G.); andrea.pession@unibo.it (A.P.); 2 Department of Medical and Surgical Sciences (DIMEC), University of Bologna, 40138 Bologna, Italy; giampaolo.ricci@unibo.it; 3 Clinica Pediatrica, Azienda Ospedaliero-Universitaria, Medicine and Surgery Department, Università di Parma, 43126 Parma, Italy; giannigiuliana90@gmail.com

**Keywords:** shellfish allergy, crustaceans, molluscs, tropomyosin, oral food challenge, allergen-specific immunotherapy

## Abstract

Shellfish is a leading cause of food allergy and anaphylaxis worldwide. Recent advances in molecular characterization have led to a better understanding of the allergen profile. High sequence homology between shellfish species and between shellfish and house dust mites leads to a high serological cross-reactivity, which does not accurately correlate with clinical cross-reactions. Clinical manifestations are immediate and the predominance of perioral symptoms is a typical feature of shellfish allergy. Diagnosis, as for other food allergies, is based on SPTs and specific IgE, while the gold standard is DBPCFC. Cross-reactivity between shellfish is common and therefore, it is mandatory to avoid all shellfish. New immunotherapeutic strategies based on hypoallergens and other innovative approaches represent the new frontiers for desensitization.

## 1. Introduction

Shellfish allergy is one of the main food allergies worldwide [1] and a leading cause of food-induced anaphylaxis [2]. This review aims to summarize the epidemiological aspects, clinical manifestations and management of shellfish allergy.

## 2. Taxonomy

Shellfish is defined as any edible marine invertebrate. Crustaceans are a subphylum of the phylum Arthropoda. Commonly consumed crustaceans, such as shrimps (or prawns, a synonym of shrimp for those of larger size and less curved), crabs, lobsters and crayfish, belong to the order Decapoda, literally “ten-footed”. Decapods have five pairs of legs on the main thoracic body along with five pairs of swimming legs on the abdomen and share a close evolutionary relationship to arachnids (dust mites, spiders, etc.) and insects (cockroaches, edible insects) [3].

Mollusks belong to the phylum Mollusca. Mollusks commonly consumed by humans belong to the classes Bivalvia, Gastropoda and Cephalopoda. Bivalves (clams, mussels) have a shell made of two systems called valves, which are generally symmetrical. Gastropods (sea slug, sea snails) are characterized by a shell without bilateral symmetry. Cephalopods (cuttlefish, squid, octopus) have a shell that is internally reduced or completely absent and a voluminous head [4].

## 3. Epidemiology

The prevalence of shellfish allergy in the Western world is approximately 0.5% [5,6,7]. Shellfish allergy often occurs in late childhood or adolescence. Therefore, the prevalence of shellfish allergy is higher in adults than in children. Moreover, the epidemiology of shellfish allergy varies across countries. Table 1 summarizes recent studies on the epidemiology of shellfish allergy. In Canada, the prevalence of shellfish allergy confirmed by IgE test and/or oral food challenge is 0.71% in adults and 0.6% in children [8], and it drops to 0.2% in children with a physician-confirmed diagnosis [9]. In the USA, shellfish allergy occurs in adulthood in 61% of patients [10], representing one of the most frequent causes of food allergy [11]. Among children with reported food allergy, shellfish allergy has a prevalence of 1.3% [12]. In Asiatic children, shellfish allergy is the commonest food allergy, due to the higher consumption of these foods [13,14]. Shellfish allergy is also very common in Brazilian children, confirming the role of food habits [15]. In European countries, self-reported prevalence of allergy to crustaceans in schoolchildren was highest in Iceland (1%), followed by Southampton and Amsterdam (0.6%), moderately common in Vilnius, Athens and Madrid (0.4–0.5%) and rare in Berlin and Lodz (0.1%–0) [16]. Challenge-proven prevalence studies are scarce. The largest oral challenge-based study showed an overall shellfish allergy prevalence of 0.3% in adults in Denmark [17].

Furthermore, it is noteworthy that shellfish is the most common trigger for foodborne anaphylaxis in Australia and in many Asia-Pacific countries [2,18,19,20,21,22]. In the USA, shellfish allergy is the most common cause of food anaphylaxis in children <6 years of age [23]. A recent study from the USA found that 37% of pediatric patients allergic to shellfish were treated with an epinephrine auto-injector in their lifetime [24]. According to a recent systematic review, shellfish-induced anaphylaxis in children has a prevalence of 0.55% in Asia (first position compared to other foods), 0.72% in North America (fifth position) and 0.11% in Europe (thirteenth position) [25].

Crustaceans are more commonly associated with allergic reactions compared to mollusks. In particular, shrimps are the crustaceans to which allergic reactions are most frequently reported [7,26]. In Europe, the prevalence of self-reported crustacean allergy varies from 0.1% in Lithuania to 5.5% in France, while oyster allergy prevalence was 1.5% in France [7,27]. Similarly, in Asia, the prevalence of allergy to crustaceans varies from 0.7% to 3.1%, while the prevalence of allergy to mollusks is 0.2% in children aged 3–7 years [28]. In Taiwan, allergy to shrimp was reported by 52% of children with food allergy aged 4-18 years, allergy to crab was reported by 33% and allergy to mollusks was reported by 14% [29]. In Brazilian children, the relative frequency of allergy to crustaceans and mollusks was similar [15].

It is unclear whether the risk of shellfish allergy may be associated with gender [10,30].

It is possible that an early introduction to the diet may reduce the prevalence of clinical allergic reactions of shellfish [31].

**Table 1:** Epidemiological data about shellfish allergy in children in different countries from 2012 to 2022. SR: self/parent report, PC: physician confirmation through skin prick tests, sIgE or oral food challenge.

Country	Year	Age (Years)	Diagnosis	Prevalence	Relative Frequency among Children with Food Allergy
Thailand [28]	2012	3–7	SR + PC		25.4% (15/59) shrimp 6.8% (4/59) crab 1.7% (1/59) squid 1.7% (1/59) mollusk
Lithuania [27]	2012	5–12	SR		2.4% (1/41) crustacean
Hong Kong [14]	2012	11–14	SR		37.8% (133/352) crustacean
Taiwan [29]	2012	0–18	SR		51.6% (1076/2086) shrimp 34% (710/2086) crab 18.4% (384/2086) mollusk
South Korea [32]	2012	0–6	SR		13.8% (86/621) crustacean
China [33]	2015	1–7	SR	4.4% (112/2540) shrimp 3.2% (81/2540) crab	
Mexico [34]	2016	5–13	SR	1.3% (12/1049) shrimp 1.3% (12/1049) other shellfish	
South Korea [32]	2017	6–16	SR	0.84% (250/29,842) crustacean	
Australia [35]	2018	10–14	SR + PC	0.3% (15/5016) shellfish	
Vietnam [36]	2019	2–6	SR + PC	3.83% (330/8620) crustacean 1.03% (88/8620) mollusk	
United States [12]	2018	0–17	SR	1.3% (499/38,408) shellfish	
Kuwait [37]	2019	11–14	SR	1.3% (48/3738) shellfish	
Europe [38]	2020	7–10	SR + PC	0.38–3.75% (64–635/16,935) shrimp	
Europe [16]	2020	6–10	SR + PC	0.2% (15/6069) crustacean	
China, Russia, India [39]	2020	6–11	SR + PC	0–1.05% (shrimps) 0.07–0.43% (crabs)	
China [40]	2020	3–5	SR + PC	0.12% (5/4151) shrimp 0.09% (4/4151) crab	
USA [24]	2021	0–18	SR + PC	0.8% (307/38,408)	
Canada [9]	2021	0–19	SR + PC	0.2% (576/288,490)	
Brazil [15]	2022	2–5	SR		31.9% (15/47) shrimp 31.9% (15/47) mollusk

## 4. Clinical Features

IgE-mediated reactions to shellfish ingestion, as with other food allergies, occur within minutes to 2 h after ingestion. Symptoms consist of skin manifestations such as urticaria–angioedema and oral allergy syndrome in 60–95% of patients, followed by gastrointestinal symptoms (nausea, vomiting, abdominal pain) in around 20% of patients, anaphylaxis in 21–33% and respiratory symptoms (rhinitis, conjunctivitis, cough, wheezing) in 5–23% [41,42,43,44]. Notably, patients with shellfish allergy often have only perioral symptoms [44,45,46].

The threshold triggering the allergic reaction depends on the patient; in fact, high thresholds of reactivity have been reported, as well as severe reactions after the intake of traces of shellfish. It has been reported that a woman with shrimp allergy developed anaphylaxis 1 min after kissing her boyfriend, who had eaten shrimp <1 h before [44,45,47]. Furthermore, shellfish has been shown to be a trigger of exercise-induced food-dependent anaphylaxis in children [48,49].

Sensitization to shellfish occurs not only by oral intake but also by skin contact and inhalation of aerosolized particles during processing or cooking. Inhalation sensitization typically occurs in workers exposed to shellfish particulates resulting from several processing activities [50,51,52]. Occupational inhalation exposure leads to rhinoconjunctivitis, nasal pruritus, asthma, coughing, urticaria, rash and hardly systemic reactions [53,54,55,56,57]. The presence of shellfish-induced occupational respiratory symptoms increases the risk of developing allergic reactions to ingested shellfish [58]. Occupational exposure may also induce contact urticaria or contact dermatitis [54,55,59].

Contrasting data on rates of tolerance development in children with shellfish allergy have been provided. Natural resolution after 5–10 years has been described in a percentage of patients ranging from 3.9% in a questionnaire study including both adults and children [60] up to 46% in a study conducted with an oral challenge in children with non-anaphylactic reactions [61]. It is unclear whether the latter findings are restricted to a subgroup of children.

## 5. Allergens

Many allergens have been characterized in several species of crustaceans and mollusks and are registered with the International Union of Immunological Societies (WHO/IUIS Allergen Nomenclature Database). The main shellfish allergens are reviewed in Table 2.

## 6. Tropomyosin

Tropomyosin is the main shellfish allergen and belongs to actin-binding proteins involved in muscle contraction [63]. Its alpha-helical coil structure results in high heat stability [64,65]. Tropomyosin is instead susceptible to degradation by gastric pepsin and trypsin [66,67]. IgE binding to purified tropomyosin has been showed in 72–98% of shellfish-allergic patients [68,69,70,71]. Tropomyosins of different crustaceans share 90–100% of sequence homology, which leads to the high degree of cross-reactivity of IgE molecular tests [72]. Tropomyosin has also been identified in mollusks, cockroaches, nematodes such as Anisakis simplex, and house dust mites [72,73,74,75]. Cross-sensitization between crustaceans and house dust mites is mainly due to Pen a 1 IgE [76].

The homology of the amino acid sequence of tropomyosin between crustaceans is 88–100%, between crustaceans and insects or mites is about 80%, between crustaceans and mollusks is 55–65% and between crustaceans and fish is 55%. The homology between mollusks ranges from 70% to 98% and between mollusks and fish is about 55%. A homology greater than 80% should be regarded as potentially cross-reactive [6].

Microarray techniques have enabled describing linear peptides involved in sensitization to allergens. To date, eight epitopes of tropomyosin have been identified [71].

## 7. Arginine Kinase

Arginine kinase (AK) is an enzyme identified in several crustaceans and a mollusk and it is involved in the regulation of cellular ATP levels [70]. Shrimp AK (Pen m 2) is the second most clinically relevant allergen following tropomyosin, as 10–20% of shellfish-allergic patients are sensitized to arginine kinase [45,77]. AK is less resistant to heat than tropomyosin [78,79]. The volatility could be responsible for symptoms induced by the inhalation of vapors [80,81].

Arginine kinase is involved in cross-reactivity between shellfish and edible insects [82].

## 8. Myosin Light Chain

Myosin light chain (MLC) is a part of the myosin macromolecular complex in muscle proteins [83]. The frequency of sensitization to this enzyme in shellfish-allergic patients varies between 19 and 55% [68,84]. Shellfish MLC is heat- and pH-stable [85]. Generally, a sensitization to MLC is found together with a sensitization to tropomyosin. However, some reports describe cases of shrimp allergy in which the shrimp MLC (Pen m 3) was the only responsible allergen [77,83,86].

## 9. Sarcoplasmic Calcium-Binding Protein

Sarcoplasmic calcium-binding protein (SCP) is a highly heat-resistant and stable protein [87,88]. Sensitization to SCP (Pen m 4) often accompanies sensitization to tropomyosin and is common in children, having been observed in up to 85% of pediatric patients [70,89].

## 10. Other Minor Allergens

Other allergens of shellfish include troponin C (Pen m 6), triose phosphate isomerase, hemocyanin, fructose biphosphate aldolase, fatty acid-binding protein, α-actinin and β-actinin, ubiquitin, paramyosin and myosin heavy chain [86]. Rates of sensitization to minor allergens are highly variable among shellfish-allergic patients and are influenced by geographic regions and age groups [68,70,77,81,84,89,90,91,92,93,94,95,96,97,98,99,100,101].

## 11. Cross-Reactivity

There is a major risk of cross-sensitization and clinical cross-reactivity between crustaceans, mollusks, house dust mites and insects [79]. Tropomyosin is the major allergen implicated in cross-reactivity [52,71,76,102,103]. The high frequency of cross-reactivity complicates the identification of the primary culprit allergen both with skin allergy testing (SPTs) and molecular testing (sIgE) [104,105,106].

Approximately 45% of individuals with a crustacean allergy are also allergic to mollusk, while 70–80% of mollusk-allergic patients also experienced allergic reactions to crustaceans [24,30,106,107,108]. Some patients may have species-specific shrimp allergy [44,109]. SPTs and serological cross-sensitization do not closely correlate with clinical cross-reactivity [43,110,111].

Tropomyosin of shellfish also shares homologies in sequence with tropomyosin of other invertebrates, including house dust mites (Der p 10). Sensitization to dust mites via inhalation has been hypothesized to secondarily trigger cross-reactivity to shellfish. This route of sensitization may also explain the late age of onset and the predominance of oral symptoms typical of shellfish allergy [72,76]. The data are supported by the high sensitization to both dust mites and shellfish in atopic populations [112,113,114,115]. On the contrary, sensitization to dust mites as cross-reaction after shellfish ingestion has also been proposed but less frequently [76,97].

Insects are largely consumed in China. In Europe, it was recently allowed to use some insects such as mealworms, crickets and locusts as foods. Patients with allergy to shrimp or house dust mites are at risk of developing clinical allergy to edible insects [116,117]. Most shrimp-allergic patients are sensitized to mealworm, although they did not previously eat it [118]. Co-sensitization to tropomyosin, arginine kinase, myosin light chain and triosephosphate isomerase probably explain cross-reactivity among insects, shrimp and mites.

There is limited evidence of cross-reactions between shellfish and fish, even though up to about 20% of adults with mollusk allergy have self-reported to be allergic to fin fish [30,119]. This coexistence may be explained by the homology between fish and shellfish tropomyosin. Overall, patients with shellfish allergy should not follow a fish-free diet [119,120].

## 12. Diagnosis

A thorough medical history, including ingested shellfish species, interval time after ingestion, symptoms, timing of resolution and treatment, is essential to properly target allergy testing. Diagnostic workup is made difficult by the wide variety of shellfish species and by cross-reactivity between shellfish. Furthermore, sensitization may be primary or secondary to cross-reactivity among homologous proteins of other invertebrates such as cockroaches and house dust mites.

Skin prick tests (SPTs) represent the first approach, being easy to perform, cheap, quick and standardized [121]. Commercial extracts have significant variability in included species and allergen content [71,122]. For this reason, SPTs may result negatively in allergic patients. Prick-by-prick with geographically relevant shellfish species showed a higher sensitivity (100%) and negative predictive value (70–100%) but lower specificity (0–41%) and positive predictive value (65–70%) than commercial SPTs (71–88%, 90–91%, 37–64%, 30–33%). The optimal decision point for SPTs has yet to be validated with larger cohorts [44,123].

The determination of serum-specific IgE could also prove useful to demonstrate sensitization. Specific IgEs are highly influenced by cross-reactivity in vitro, without clinical correlates. A negative predictive value of IgE to shrimps (91.3%) is comparable to that of SPTs (90%), while a positive predictive value seems higher (41–71% for IgE, 33.3% for SPTs) but still inaccurate [69,123].

Molecular diagnosis may be a promising approach to increase in vitro diagnostic accuracy [68,84,109,124]. Tropomyosin sensitization is critical in the diagnosis of shellfish allergy although other allergens can be involved [71]. Two shrimp tropomyosin are commercialized, Pen a 1 and Pen m 1. Shrimp tropomyosin (Pen m 1) is the major allergen in shrimp-sensitized patients. However, the clinical significance of positive IgE to Pen m 1 is unclear since the frequency of sensitization to Pen m 1 ranged from 34% to 63% in populations with shrimp allergy from Hong Kong, Thailand, Japan, Brazil, Spain and Italy [84,109,125,126,127].

In Hong Kong, in patients with shrimp allergy, the area under the curve of shrimp SPTs was 0.74, that of specific IgE to shrimp was 0.75, that of Pen m 1-sIgE was 0.70, that of Pen m 4-sIgE was 0.77, that of Pen m 6-sIgE was 0.78, that of Pen m 13-sIgE (fatty acid-binding protein) was 0.77 and that of Pen m 14-sIgE (glycogen phosphorylase) was 0.59; the areas under the curve of the same IgEs in Thailand were 0.7, 0.7, 0.89, 0.96, 0.86, 0.81 and 0.54, respectively [128]. While for other food allergies, a link between specific epitopes and allergic reactivity (for example persistent or more severe allergy) has been found, in shellfish allergy, the clinical significance of different allergens is not yet clear. Also, the recognition of epitopes differs between children and adults [70].

Basophil activation test for shrimp reached an area under the curve of 0.88 in a Chinese population with shrimp allergy [125].

A recent study first assessed the diagnostic value of the nasal allergen provocation test (NAPT) in shellfish allergy diagnosis, showing that it differentiated between shrimp-allergic and -tolerant subjects with high sensitivity (90%) and specificity (89%) [86].

The use of the ExiLe technology is also under study. ExiLe is based on a rat basophilic leukemia cell line transfected with the a/b/g subunits of the human IgE receptor FceRI and the luciferase reporter gene (RS-ATL8). The test is based on the idea that the measurement of luciferase’s signal reflects the degree of IgE crosslinking [129]. ExiLe technology has been promisingly demonstrated to have better diagnostic accuracy than SPTs and sIgE [125,130].

Oral food challenge remains the gold standard for confirming the diagnosis of food allergies, although it is time-consuming and expensive and carries the risk of severe allergic reactions. An initial dose of 3 mg of shrimp proteins has been proposed. The dose is then increased every 15 to 30 min [131,132,133]. The protocol should be individualized to achieve the recommended daily dose based on the age of the patient. On average, about 0.1–1.0 g of shellfish pulp must be ingested to trigger an allergic response [111].

The gold standard is the double-blind placebo-controlled food test (DBPCFC). In clinical practice, tests are usually performed open unless there are diagnostic doubts.

## 13. Differential Diagnosis

Non-IgE-mediated forms of shellfish allergy, mainly represented by food protein-induced enterocolitis syndrome (FPIES), are also described [134]. Clinical manifestations of FPIES are different from IgE-mediated symptoms and consist of profuse vomiting, typically 1–3 h after ingestion, diarrhea, pallor, hypothermia and hypotension or flaccidity.

Adverse reactions to shellfish may also be caused by non-immunologic mechanisms. As filter feeders, these shellfish may accumulate bacteria (e.g., Vibrio, Klebsiella), viruses (e.g., Hepatitis A) and toxins produced by algae (shellfish poisoning syndromes). Shellfish may also be infested by Anisakis, a parasitic nematode mainly found in fish, but also in large crustaceans and cephalopods. The parasite is ingested while eating raw or undercooked seafood, then fails to reproduce in the human host and dies in about 3 weeks. The acute gastric form, occurring 2–8 h after the ingestion, consists of diffuse epigastric pain, fever, nausea and vomiting. Chronic intestinal anisakiasis occurs 5-7 days after ingestion, following the attachment of the larvae to the intestinal mucosa with the formation of granulomas and abscesses in the intestinal wall. Patients previously exposed to Anisakis may also develop hypersensitivity to worm antigens with the development of allergic reactions or anaphylaxis upon re-exposure to live or dead larvae [111,135,136,137,138,139,140,141,142,143,144]. Different diagnoses of shellfish allergy are reviewed in Table 3.

## 14. Management

Management of shellfish allergy is based on avoidance of shellfish and treatment with rescue medication in case of an allergic episode. Some patients with shellfish allergy can tolerate particular crustaceans or mollusks. However, cross-reactivity is frequent, so patients with crustacean or mollusk allergy should avoid all shellfish species to which they are sensitized also because of contamination risk, unless tolerance is demonstrated by food challenge. Contact with shellfish cooking vapors should also be avoided. In case of severe reactions, the prescription of adrenaline autoinjectors with a personalized emergency action plan is recommended.

The goal of novel therapies for food allergy is to desensitize patients and restore food tolerance in order to improve patients’ quality of life. Allergen-specific immunotherapy (AIT) involves the administration of gradually increasing amounts of allergen extracts to induce desensitization. AIT for shrimp has not yet been introduced in clinical practice. Preliminary data suggest that it is safe and well tolerated [145]. The main problem with this approach is that shrimp extracts are heterogeneous in allergen content, leading to diverse responses. A recent study reported the safety and efficacy of omalizumab-facilitated oral immunotherapy for shrimp allergy [146]. Allergen-specific AIT with tropomyosin has been studied in a murine model, showing successful desensitization, but with potentially serious side effects [147].

Since tropomyosin is highly allergenic, research in shellfish immunotherapy has also focused on the development of hypoallergenic variants of tropomyosin to improve the safety of AIT. Hypoallergenic tropomyosin is obtained through methods including enzymatic crosslinking, polypeptide fragmentation and epitope manipulation [130,148,149].

AIT with linear peptides corresponding to T-cell epitopes is also under study [150,151,152]. A recently proposed therapeutic approach is based on T-cell epitopes and CpG-ODN agonist of Toll-like receptor 9 (TLR9) in nanoparticles. CpG-ODN is a TLR9 ligand known to downregulate the established Th2 response and induce Th1 immunity [153,154].

The correlation between shellfish and dust mites suggests the possibility that AIT for dust mites may lead to the improvement of shellfish allergy [155,156]. On the other hand, cases have been reported of shrimp allergy following mite-specific immunotherapy [157,158,159].

Anti-IgE therapies such as omalizumab and anticytokine drugs are traditional nonspecific treatments that can be used alone or in combination with AIT for desensitization.

## 15. Conclusions

Shrimp allergy is an increasing worldwide problem affecting not only adults but also children, and is a frequent cause of anaphylaxis. Molecular characterization may potentially allow a better description of shellfish allergenic profiles. The clinical relevance of the different allergens and epitopes remains to be determined and could lead to improvements in diagnostic and therapeutic approaches. More reliable and specific diagnostic tools that correlate with clinical reactivity are needed. New immunotherapeutic strategies based on hypoallergens and other innovative approaches represent the new frontiers for desensitization.

## Figures and Tables

**Table 2:** Crustacea allergens and Mollusca allergens (WHO/IUIS Allergen Nomenclature Sub-Committee) [62].

Common Name	Scientific Name	Allergen	Protein Type	Molecular Weight (kDa)
**a. Crustacea allergens**				
**SHRIMP**				
North Sea shrimp	Crangon crangon	Cra c 1	Tropomyosin	38
		Cra c 2	Arginine kinase	45
		Cra c 4	Sarcoplasmic calcium-binding protein	25
		Cra c 5	Myosin light chain 1	17.5
		Cra c 6	Troponin C	21
		Cra c 8	Triosephosphate isomerase	28
White shrimp	Litopenaeus vannamei	Lit v 1	Tropomyosin	36
		Lit v 2	Arginine kinase	40
		Lit v 3	Myosin light chain 2	20
		Lit v 4	Sarcoplasmic calcium-binding protein	20
		Lit v 13	Fatty acid-binding protein	15
Black tiger shrimp	Penaeus monodon	Pen m 1	Tropomyosin	38
		Pen m 2	Arginine kinase	34
		Pen m 3	Myosin light chain 2	20
		Pen m 4	Sarcoplasmic calcium-binding protein	20
		Pen m 6	Troponin C	16.8
		Pen m 7	Hemocyanin	76
		Pen m 8	Triosephosphate isomerase	27
		Pen m 13	Cytoplasmic fatty acid-binding protein	20
		Pen m 14	Glycogen phosphorylase-like protein	95
Brown shrimp	Penaeus aztecus	Pen a 1	Tropomyosin	36
Shrimp	Penaeus indicus	Pen i 1	Tropomyosin	34
White-legged freshwater shrimp	Exopalaemon modestus	Exo m 1	Tropomyosin	38
Shrimp	Metapenaeus ensis	Met e 1	Tropomyosin	34
Northern shrimp	Pandalus borealis	Pan b 1	Tropomyosin	37
**CRAB**				
Mud crab	Scylla paramamosain	Scy p 1	Tropomyosin	38
		Scy p 2	Arginine kinase	40
		Scy p 3	Myosin light chain	18
		Scy p 4	Sarcoplasmic Ca^+^ binding protein	20
		Scy p 8	Triosephosphate isomerase	28
		Scy p 9	Filamin C	90
Warrior swimming brown crab	Callinectes bellicosus	Cal b 2	Arginine kinase	40
Crab	Charybdis feriatus	Cha f 1	Tropomyosin	34
Chinese mitten crab	Eriocheir sinensis	Eri s 2	Ovary development-related protein	28.2
Blue swimmer crab	Portunus pelagicus	Por p 1	Tropomyosin	39
**LOBSTER**				
American lobster	Homarus americanus	Hom a 1	Tropomyosin	34
		Hom a 3	Myosin light chain 2	23
		Hom a 6	Troponin C	20
Spiny lobster	Panulirus stimpsoni	Pan s 1	Tropomyosin	34
**b. Mollusca allergens**				
**BIVALVIA**				
Pacific oyster	Crassostrea gigas	Cra g 1	Tropomyosin	38
Pacific oyster	Crassostrea angulata	Cra a 2	Arginine kinase	38
Cockle	Fulvia mutica	Cra a 4	Sarcoplasmic calcium-binding protein	20–25
Sydney rock oyster	Saccostrea glomerata	Sac g 1	Tropomyosin	38
**GASTEROPODA**				
Brown garden snail	Helix aspersa	Hel as 1	Paramyosin	99
Veined rapa whelk	Rapana venosa	Rap v 2	Paramyosin	
**CEFALOPODA**				
Perlemoen abalone	Haliotes midae	Hal m 1	Tropomyosin	49
Jade tiger abaolone	Haliotis laevigata, Haliotis rubra	Hal l 1	Tropomyosin	33.4
Japanese flying squid	Todarodes pacificus	Tod p 1	Tropomyosin	38

**Table 3:** Different diagnoses of shellfish.

Name	Affected Shellfish	Cause	Onset (h after Ingestion)	Clinical Findings
IMMUNOLOGICAL REACTIONS				
IgE-mediated shellfish allergy	Crustaceans and mollusks	IgE-mediated adverse reaction to shellfish	Minutes–4 h	Oral allergy syndrome, urticaria, rhinitis, nausea, vomiting, anaphylaxis
Food Protein-Induced Enterocolitis Syndrome (FPIES)	Crustaceans and mollusks	T-cell-mediated intestinal inflammation (not clearly understood pathogenesis)	1–4 h	Profuse vomiting, diarrhea, sepsis-like picture
Anisakis allergy	Crustaceans and mollusks	IgE-mediated adverse reaction to Anisakis infesting seafood	2–24 h	Urticaria, angioedema, abdominal pain, anaphylaxis
SHELLFISH CONTAMINATION				
Staphylococcus aureus food poisoning	Crustaceans and mollusks	Ingestion of fish contaminated by hands at room temperature	1–6 h	Nausea, vomiting, abdominal pain, fever
Bacterial or viral contamination (e.g., Vibrio Cholerae, Hepatitis A)	Crustaceans and mollusks	Ingestion of raw fish harvested in contaminated waters	2–24 h	Nausea, vomiting, diarrhea, abdominal pain, fever
Anisakiasis	Crustaceans and mollusks	Ingestion of raw, undercooked or pickled fish with alive parasites (Anisakis)	2–24 h	Nausea, vomiting, abdominal pain
SHELLFISH POISONING				
Paralytic shellfish poisoning	Bivalve mollusks	Saxitoxin formed by algae	1–2 h	Paresthesias, dizziness, ataxia
Neurotoxic shellfish poisoning	Bivalve mollusks	Brevetoxin formed by algae	3–4 h	Nausea, vomiting, diarrhea, abdominal pain Paresthesias, dizziness, ataxia rhinorrhea, bronchoconstriction
Diarrhetic shellfish poisoning	Bivalve mollusks	Okadaic acid formed by algae	1–15 h	Nausea, vomiting, diarrhea, abdominal pain
Amnesic shellfish poisoning	Bivalve mollusks	Domoic acid formed by algae	24–48 h	Disorientation, amnesia, headache, diarrhea, abdominal pain

## Data Availability

Not applicable.
